# Exploring the bioactive potential of bovine pericardium membrane combined with hyaluronic Acid: characterization and cellular viability analyses

**DOI:** 10.1016/j.bbrep.2026.102490

**Published:** 2026-02-06

**Authors:** Dwi Wahyu Indrawati, Ernie Maduratna Setiawatie, Retno Pudji Rahayu, Rizky Briliant Syah Manurung, Mohammed Ahmed Aljunaid

**Affiliations:** a Doctor Program of Dental Health Sciences, Faculty of Dental Medicine, Universitas Airlangga, Surabaya, Indonesia; b Department of Dentistry, Universitas Muhammadiyah Sidoarjo, Indonesia; c Department of Periodontic, Faculty of Dental Medicine, Universitas Airlangga, Surabaya, Indonesia; d Department of Oral Pathology and Maxillofacial, Faculty of Dental Medicine, Universitas Airlangga, Surabaya, Indonesia; e Master Program of Dental Health Sciences, Faculty of Dental Medicine, Universitas Airlangga, Surabaya, Indonesia; f School of Oral and Dental Medicine, Faculty of Medicine, Taiz University, Taiz, Yemen; g Faculty of Oral and Dental Medicine, Al-Saeed University, Taiz, Yemen

**Keywords:** Bovine pericardium membrane, Hyaluronic acid, Biomaterials, Tissue regeneration, Fibroblast viability

## Abstract

This study explores the bioactive potential of bovine pericardium membranes combined with hyaluronic acid (HA, 120 kDa) at concentrations of .5%, 1.0%, and 2.0% through physicochemical characterization and fibroblast cell viability analysis. A laboratory experimental design was employed to evaluate bovine pericardium membranes modified with hyaluronic acid at different concentrations. Material characterization was conducted employing Fourier Transform Infrared Spectroscopy (FTIR), Scanning Electron Microscopy (SEM), and X-ray Diffraction (XRD) analyses. Fibroblast cell viability was assessed using the MTT assay, and statistical analysis was conducted using the ANOVA test. The results demonstrated that hyaluronic acid modification altered the physicochemical characteristics of the membrane, including increased surface hydrophilicity. The cell viability test revealed comparable fibroblast viability among HA-modified membranes, indicating no cytotoxic effects. FTIR and SEM analyses confirmed chemical interactions and morphological features associated with favorable cell–material interactions. These findings suggest that bovine pericardium membrane with hyaluronic acid 120 kDa exhibit favorable physicochemical characteristics and in vitro cytocompatibility, indicating their potential for tissue engineering applications. This research provides a scientific foundation for developing innovative biomaterials that support favorable cell–material interactions for tissue regeneration research, contributing to advancements in tissue regeneration therapy.

## Highlights

• Bovine pericardium membranes were modified with hyaluronic acid (120 kDa).
• Hyaluronic acid incorporation altered membrane physicochemical characteristics.
• FTIR and SEM analyses confirmed preserved collagen structure and surface morphology.
• HA-modified membranes showed comparable fibroblast viability with no cytotoxic effects.
• The membranes demonstrate potential as biocompatible biomaterials for tissue engineering applications.

## 1 Introduction

Collagen-based biomaterials, such as **the** bovine pericardium membrane have been widely used in clinical applications due to their good mechanical properties and biocompatibility. This material is often utilized in tissue regeneration, but the limitations of its bioactive properties are a major obstacle in supporting cell adhesion and proliferation [1]. In recent decades, the development of biomaterials capable of supporting optimal tissue regeneration has become an important focus in the biomedical field. One potential innovative approach is to modify these membranes with bioactive compounds [2].

Hyaluronic acid, which is a natural polysaccharide in the extracellular matrix, has the ability to increase the bioactivity of biomaterials. Hyaluronic acid is known to support cell migration, adhesion and proliferation, while also playing a role in accelerating the wound healing process [3]. The molecular weight of hyaluronic acid also affects its performance where hyaluronic acid with a low molecular weight, such as 120 kDa, has an advantage in maintaining tissue structure and reducing inflammation. The combination of bovine pericardium membrane with low molecular weight hyaluronic acid is expected to improve its bioactive properties, making it more effective for tissue regeneration applications [4]; [5].

This study aimed to evaluate the physicochemical characteristics and fibroblast viability of bovine pericardium membranes modified with hyaluronic acid (120 kDa) at different concentrations (.5%, 1.0%, and 2.0%). The main focus of the study is to characterize physicochemical properties and analyze the viability of fibroblast cells on the modified membrane. The results of this research are expected to provide a scientific basis for the development of more innovative and effective regenerative materials [6].

The main hypothesis of this study is that the combination of bovine pericardium membrane with hyaluronic acid of 120 kDa can increase hydrophilicity, improve surface morphology, and to support fibroblast viability and maintain cytocompatibility [7]. To test this hypothesis, this study integrates physicochemical characterization methods such as morphological analysis, hydrophilicity, and thermal stability, as well as fibroblast cell viability tests with the MTT method [8].

Previous studies have shown that bovine pericardium membranes have the potential to be used as scaffolds in tissue regeneration due to their collagen content. However, the limitations of its bioactive properties are a challenge that needs to be overcome. Another study indicates that the addition of hyaluronic acid can improve the bioactive properties of biomaterials, especially through increased hydrophilicity and cell adhesion [9]. However, specific studies on the combination of bovine pericardium membrane and low-molecular-weight hyaluronic acid such as 120 kDa, are still rare. Therefore, this study aims to fill this scientific gap by exploring the bioactive potential of this combination of materials [10]; [11].

## 2 Material methods

### 2.1 Research design

This study used a laboratory experimental research design to evaluate the bioactive potential of bovine pericardium membrane modified with hyaluronic acid (120 kDa) at concentrations of .5%, 1.0%, and 2.0%. A quantitative approach is used to analyze changes in the physicochemical properties of membranes as well as the viability of fibroblast cells through various standardized test methods [6].

### 2.2 Subjects and samples

The main sample in this study is the bovine pericardium membrane obtained through the process of decalcification and sterilization. In addition, fibroblast cell cultures are used as biological models to test membrane biocompatibility and viability. The samples consisted of bovine pericardium membranes modified with hyaluronic acid (120 kDa) at concentrations of .5%, 1.0% and 2.0% [12]

### 2.3 Data collection methods

Data is collected through several stages:

a. Physicochemical Characterization:
  - The surface morphology was analyzed using electron microscopy (SEM).
  - Identifying functional groups and chemical interactions between pericardium membrane bovine and hyaluronic acid using Fourier Transform Infrared Spectroscopy (FTIR) [13].
  - Determining changes in the crystallinity structure of the membrane due to X-Ray Diffraction (XRD) [14].
b. Cell Viability Test:

The viability of fibroblasts was assessed using the MTT assay after 7 days of incubation on hyaluronic acid–modified bovine pericardium membranes [15].

### 2.4 Data analysis

The data from physicochemical characterization were analyzed descriptively to compare changes in membrane properties before and after modification. Cell fibroblast viability data from MTT tests were analyzed quantitatively using one-way ANOVA, to evaluate the significance of differences in fibroblast viability among the different hyaluronic acid concentrations. All analyses are carried out with the help of statistical software to ensure the validity and reliability of the research results [16].

## 3 Results

### 3.1 Fibroblast viability test

Fibroblast viability on bovine pericardium membranes combined with hyaluronic acid (HA) at different concentrations was evaluated using the MTT assay. The quantitative viability results are presented in Table 1. Data are presented as mean values derived from five independent replicates. As shown in Table 1, fibroblast viability in the group treated with 120 kDa hyaluronic acid at .5% combined with bovine pericardium membrane showed a mean viability of 84.915%. Comparable mean fibroblast viability values were observed across all HA-modified membrane groups**. No statistically significant differences were detected among the tested HA concentrations** and the 2.0% HA-modified membrane group demonstrated a mean viability of 85.018%. Cell-only and medium-only conditions were included to validate the MTT assay performance, showing 100% and 0% viability, respectively.

**Table 1:** Fibroblast viability test from bovine pericardium membrane and hyaluronic acid.

Replication	Media Control	Cell Control	120 kDa .5% + Bovine Pericardium Membrane	120 kDa 1.0% + Bovine Pericardium Membrane	120 kDa 2.0% + Bovine Pericardium Membrane
1	0	100	88.124	84.830	83.533
2	0	100	76.127	85.977	82.471
3	0	100	86.574	89.887	83.435
4	0	100	89.067	81.056	88.030
5	0	100	84.684	89.047	87.622
**Mean**	**0**	**100**	**84.915**	**86.159**	**85.018**
**Standard Deviation**	**0**	**0**	**5.190**	**3.540**	**2.600**

Statistical normality testing using the Kolmogorov–Smirnov and Shapiro–Wilk tests indicated that all HA-treated groups followed a normal distribution (p > 0.05), as summarized in Table 2. Homogeneity of variance analysis using Levene's test further confirmed that the data were homogeneous across groups (p = 0.622), as shown in Table 3. Based on these assumptions, one-way ANOVA was performed to compare fibroblast viability among the three HA-modified membrane groups. The analysis revealed no statistically significant difference in mean viability between groups (F = .155, p = 0.858), as presented in Table 4.

**Table 2:** Tests of normality.

	Group	Kolmogorov-Smirnova			Shapiro-Wilk		
		Statistics	Df	Sig.	Statistics	Df	Sig.
Viability	120 kDa .5% + Bovine Pericardium Membrane	.282	5	.200∗	.827	5	.132
	120 kDa 1.0% + Bovine Pericardium Membrane	.193	5	.200∗	.947	5	.717
	120 kDa 2.0% + Bovine Pericardium Membrane	.316	5	.115	.818	5	.114

**Table 3:** Test of homogeneity of variances.

		Levene Statistic	df1	DF2	Sig.
Viability	Based on Mean	.495	2	12	.622
	Based on Median	.257	2	12	.778
	Based on Median and with adjusted df	.257	2	8.144	.780
	Based on trimmed mean	.442	2	12	.653

**Table 4:** Anova test.

ANOVA					
	Sum of Squares	Df	Mean Square	F	Sig.
Between Groups	4.768	2	2.384	.155	.858
Within Groups	184.701	12	15.392		
Total	189.469	14			

Post hoc multiple comparison analysis using Tukey's HSD test confirmed that there were no significant pairwise differences between the .5%, 1.0%, and 2.0% HA-modified membrane groups (p > 0.05 for all comparisons), as detailed in Table 5. Overall, the results demonstrate that bovine pericardium membranes combined with hyaluronic acid at concentrations ranging from .5% to 2.0% exhibit comparable fibroblast viability, indicating good biocompatibility of the membranes regardless of HA concentration.

**Table 5:** Multiple comparisons.

Multiple Comparisons						
Dependent Variable: Viability						
Tukey HSD						
(I) Group	(J) Group	Mean Difference (I-J)	Std. Error	Sig.	95% Confidence Interval	
					Lower Bound	Upper Bound
120 kDa .5% + Bovine Pericardium Membrane	120 kDa 1.0% + Bovine Pericardium Membrane	−1.244200	2.481271	.872	−7.86389	5.37549
	120 kDa 2.0% + Bovine Pericardium Membrane	−.103000	2.481271	.999	−6.72269	6.51669
120 kDa 1.0% + Bovine Pericardium Membrane	120 kDa .5% + Bovine Pericardium Membrane	1.244200	2.481271	.872	−5.37549	7.86389
	120 kDa 2.0% + Bovine Pericardium Membrane	1.141200	2.481271	.891	−5.47849	7.76089
120 kDa 2.0% + Bovine Pericardium Membrane	120 kDa .5% + Bovine Pericardium Membrane	.103000	2.481271	.999	−6.51669	
	120 kDa 1.0% + Bovine Pericardium Membrane	−1.141200	2.481271	.891	−7.76089	

### 3.2 FTIR test

**Fig. 1:**
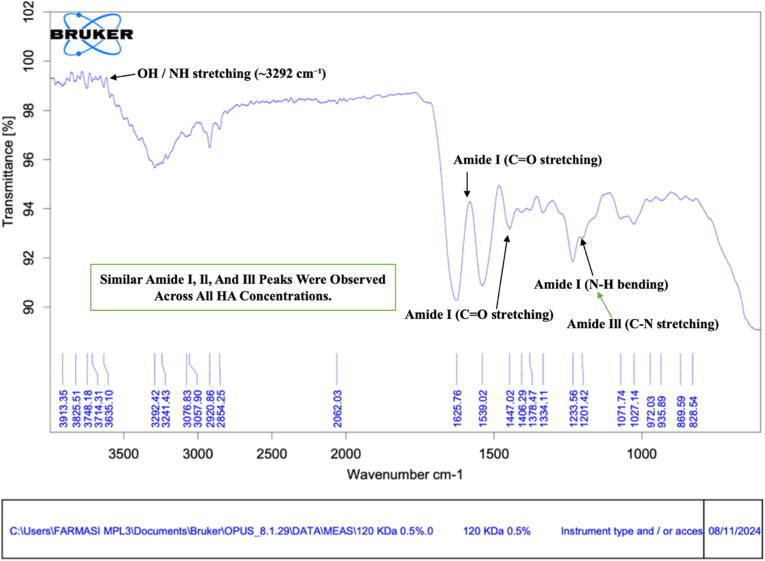
FTIR spectra of bovine pericardium membranes combined with hyaluronic acid (120 kDa) at a concentration of .5%. **Arrows indicate characteristic absorption peaks corresponding to amide I, amide II, amide III, and hydroxyl (–OH) functional groups.**

**Fig. 2:**
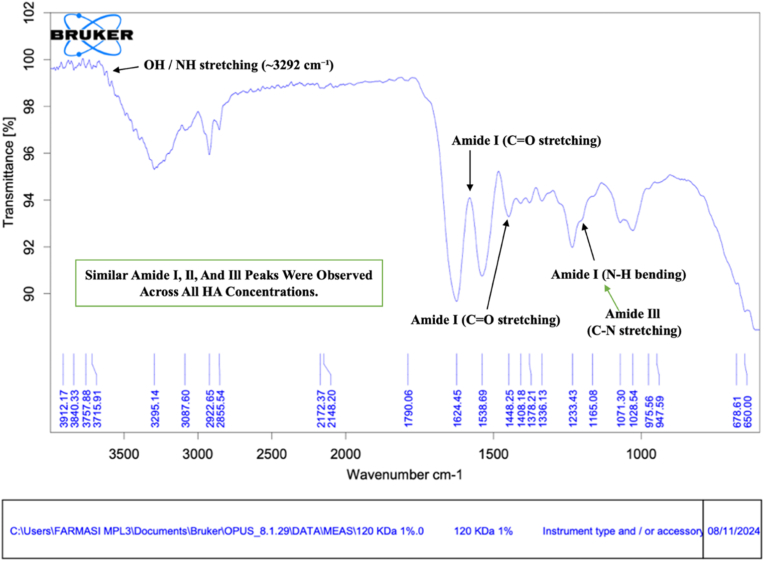
FTIR spectra of bovine pericardium membranes combined with hyaluronic acid (120 kDa) at a concentration of 1.0%. **Arrows indicate characteristic absorption peaks corresponding to amide I, amide II, amide III, and hydroxyl (–OH) functional groups.**

**Fig. 3:**
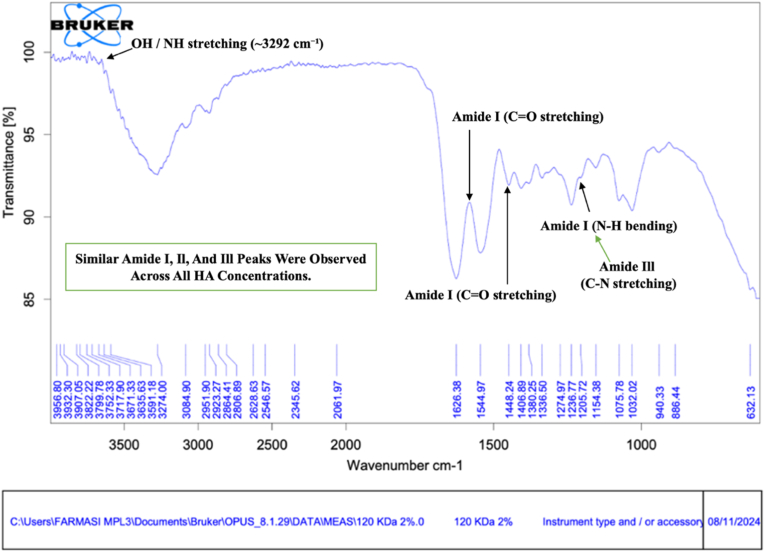
FTIR spectra of bovine pericardium membranes combined with hyaluronic acid (120 kDa) at a concentration of 2.0%. **Arrows indicate characteristic absorption peaks corresponding to amide I, amide II, amide III, and hydroxyl (–OH) functional groups.**

For clarity, characteristic FTIR peaks, including amide I, amide II, amide III, and hydroxyl groups, are highlighted using arrows in all spectra. The Fourier Transform Infrared (FTIR) spectra of bovine pericardium membranes modified with 120 kDa hyaluronic acid (HA) at concentrations of .5%, 1.0%, and 2.0% are presented in Fig. 1, Fig. 2, Fig. 3, respectively.

As shown in Fig. 1, the FTIR spectrum of the membrane combined with .5% HA exhibited a broad absorption band at approximately 3292 cm^−1^, corresponding to hydroxyl (–OH) stretching vibrations, which are characteristic of collagen and hydrogen-bonded water molecules. Peaks observed at around 2924 cm^−1^ were assigned to C–H stretching vibrations of methylene and methyl groups, commonly found in biological tissues. The presence of prominent amide I (≈1640 cm^−1^), amide II (≈1539 cm^−1^), and amide III (≈1233 cm^−1^) bands confirmed the preservation of collagen protein structures within the bovine pericardium membrane after HA incorporation.

The FTIR spectrum of the membrane modified with 1.0% HA (Fig. 2) demonstrated a similar spectral profile, with a slightly increased intensity of the hydroxyl (–OH) stretching band at approximately 3295 cm^−1^. The characteristic collagen-associated amide I (≈1640 cm^−1^), amide II (≈1548 cm^−1^), and amide III (≈1233 cm^−1^) bands remained clearly detectable. In addition, peaks corresponding to C–O stretching vibrations around 1071 cm^−1^ were observed, indicating the contribution of polysaccharide-related functional groups from hyaluronic acid [16].

As shown in Fig. 3, the membrane combined with 2.0% HA also exhibited the typical collagen-related amide I (≈1640 cm^−1^), amide II (≈1545 cm^−1^), and amide III (≈1237 cm^−1^) bands. The hydroxyl stretching band around 3274 cm^−1^ showed comparable intensity to that observed in the 1.0% HA group, suggesting saturation of hydroxyl group incorporation at higher HA concentration. Additional C–O stretching vibrations around 1076 cm^−1^ further supported the successful incorporation of hyaluronic acid into the membrane matrix [11].

Overall, the FTIR spectra across all HA concentrations demonstrated consistent preservation of collagen structures within the bovine pericardium membrane. The gradual increase and subsequent stabilization of hydroxyl- and C–O-related bands with increasing HA concentration indicate the incorporation of hyaluronic acid within the membrane matrix without altering the fundamental protein backbone [17].

### 3.3 X-ray diffraction (XRD)

**Fig. 4:**
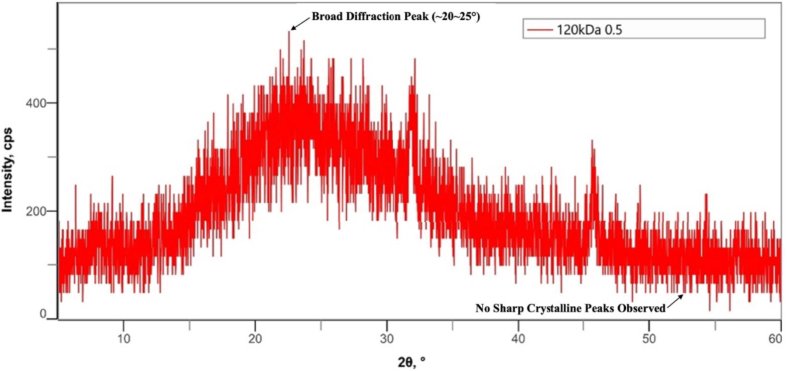
Combination of Bovine Pericardium Membrane and Hyaluronic Acid (120 kDa) at a concentration of .5%.

**Fig. 5:**
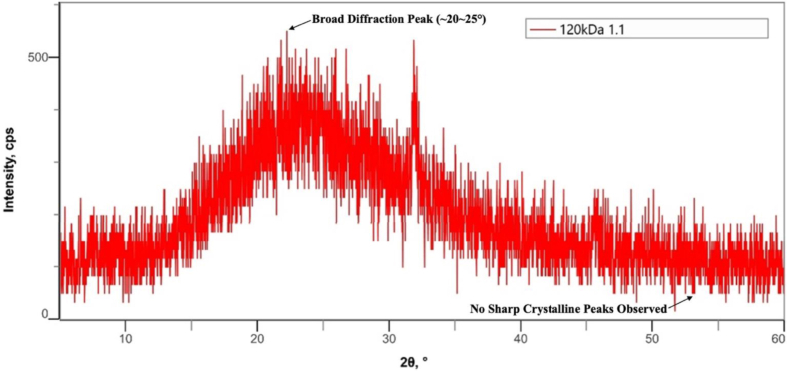
Combination of Bovine Pericardium Membrane and Hyaluronic Acid (120 Kda) at a concentration of 1.0%.

**Fig. 6:**
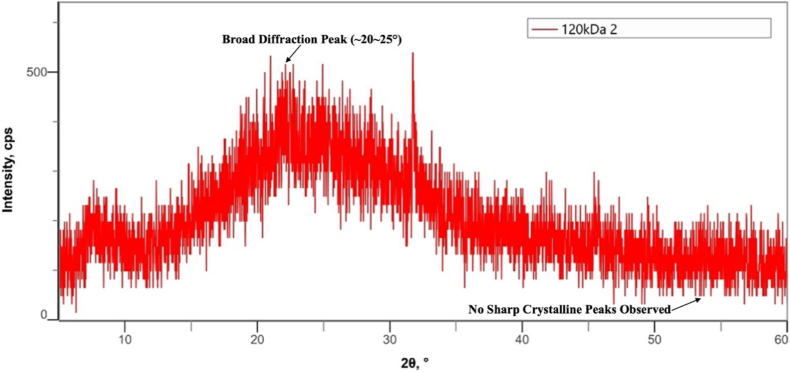
Combination of Bovine Pericardium Membrane and Hyaluronic Acid (120 Kda) at a concentration of 2.0%.

The X-ray diffraction (XRD) patterns of bovine pericardium membranes modified with 120 kDa hyaluronic acid (HA) at concentrations of .5%, 1.0%, and 2.0% are presented in Fig. 4, Fig. 5, Fig. 6, respectively.

As shown in Fig. 4, the bovine pericardium membrane combined with .5% HA exhibited a broad diffraction halo in the 2θ range of approximately 20°–30°, which is characteristic of an amorphous structure. No sharp diffraction peaks corresponding to crystalline phases were detected, indicating that the incorporation of hyaluronic acid at this concentration did not induce crystallization within the membrane matrix.

Similarly, the XRD pattern of the membrane modified with 1.0% HA (Fig. 5) demonstrated a predominantly amorphous profile, with a broad diffuse peak centered around 2θ = 20°–30°. The absence of distinct crystalline reflections indicates that increasing the HA concentration to 1.0% did not significantly alter the amorphous nature of the bovine pericardium membrane [18].

As presented in Fig. 6, the membrane combined with 2.0% HA also displayed an amorphous diffraction pattern, characterized by a wide halo in the same angular range. No additional crystalline peaks were observed at higher HA concentration, confirming that hyaluronic acid incorporation up to 2.0% did not affect the phase structure of the membrane [19,20].

Across all HA concentrations, the XRD results demonstrated consistent amorphous characteristics, with no evidence of crystalline phase formation. Due to the absence of sharp diffraction peaks, crystallite size could not be determined using conventional XRD analysis [21,22].

### 3.4 Scanning electron microscopy (SEM)

**Fig. 7:**
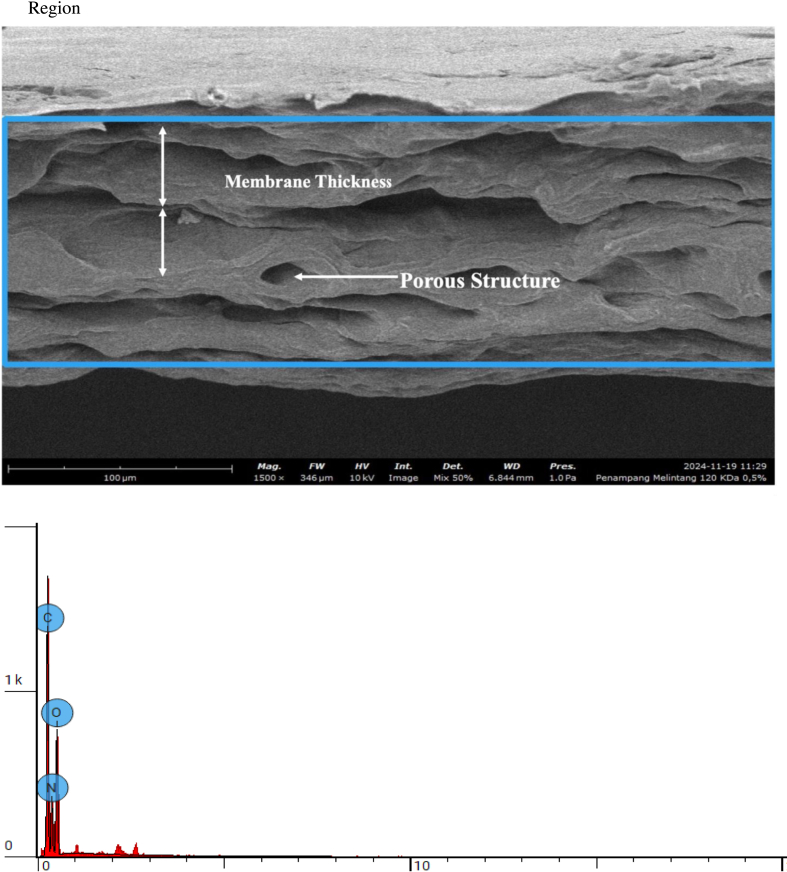
Cross section 120 KDa .5%.

**Fig. 8:**
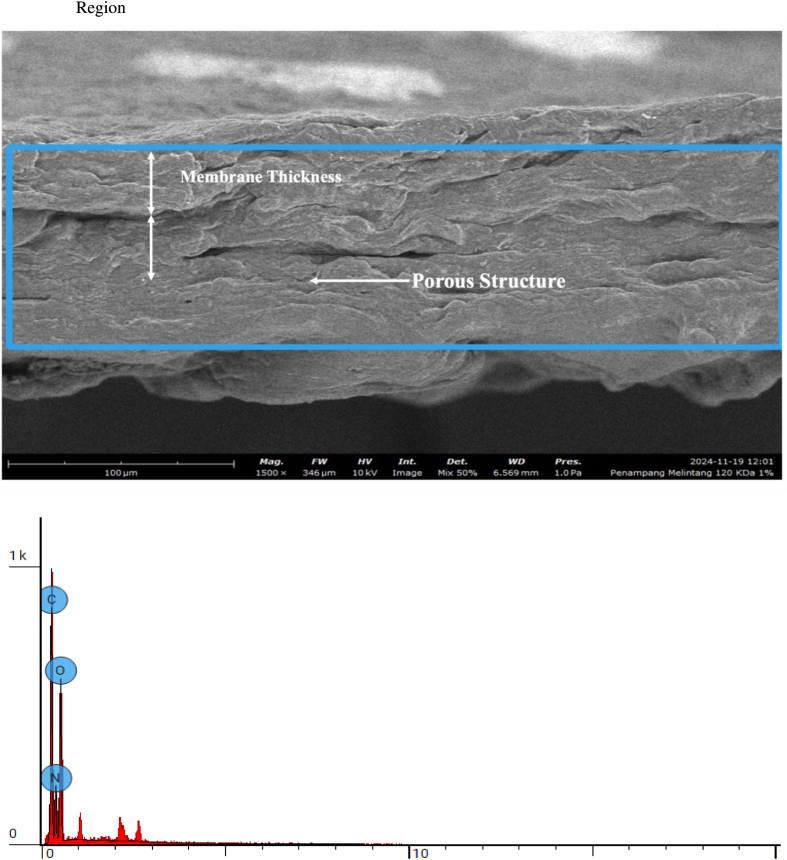
Cross section 120 KDa 1.0%.

**Fig. 9:**
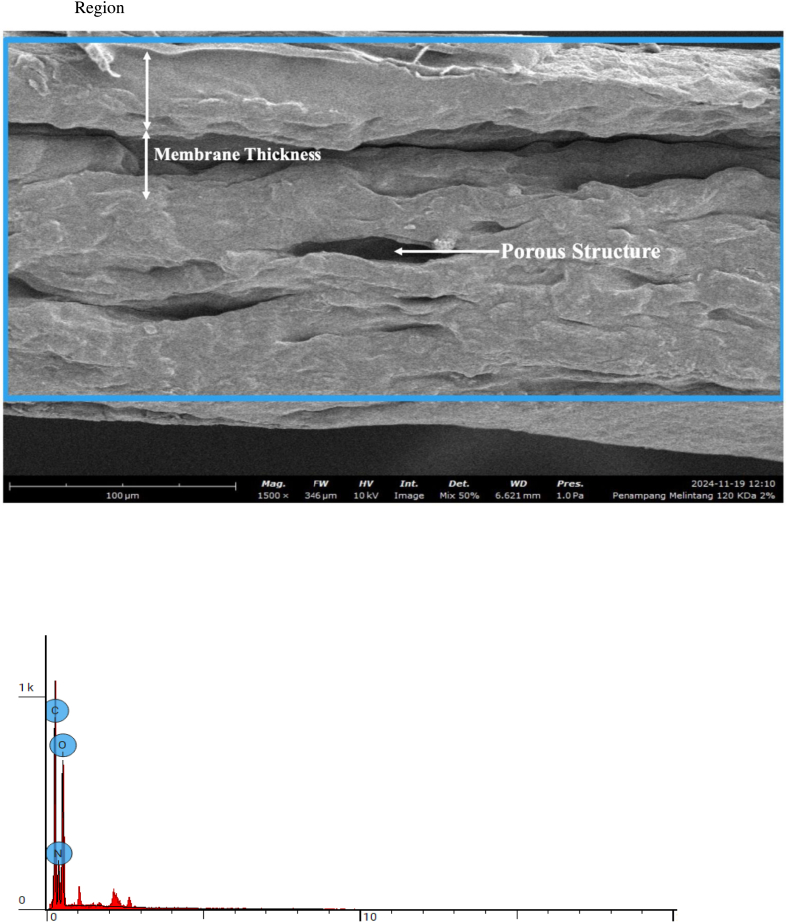
Cross section: 120 KDa 2.0%.

Cross-sectional morphology and elemental composition of bovine pericardium membranes modified with 120 kDa hyaluronic acid (HA) at concentrations of .5%, 1.0%, and 2.0% were evaluated using scanning electron microscopy coupled with energy-dispersive X-ray spectroscopy (EDS). The SEM images and corresponding elemental analyses are presented in Fig. 7, Fig. 8, Fig. 9, respectively.

As shown in Fig. 8, the cross-sectional SEM image of the membrane combined with .5% HA revealed a compact and homogeneous structure without visible cracks or delamination. Elemental analysis demonstrated that the membrane was primarily composed of carbon (C), nitrogen (N), and oxygen (O), with atomic concentrations of 36.33%, 35.10%, and 28.57%, respectively.

The membrane modified with 1.0% HA (Fig. 8) exhibited a similar cross-sectional morphology, maintaining structural continuity across the membrane thickness. Elemental analysis showed a slight reduction in carbon and nitrogen content, with atomic concentrations of 34.67% (C) and 32.32% (N), accompanied by an increase in oxygen content to 33.01%.

As presented in Fig. 9, the membrane combined with 2.0% HA also displayed a dense and uniform cross-sectional morphology. Elemental analysis revealed a further increase in oxygen content, reaching an atomic concentration of 34.83%, while carbon and nitrogen concentrations slightly decreased to 34.98% and 30.19%, respectively.

Across all HA concentrations, the SEM images demonstrated consistent membrane integrity and homogeneity, while elemental analysis revealed an increase in oxygen content with increasing HA concentration. No additional elemental contaminants were detected, as only C, N, and O were identified in all samples.

## 4 Discussion

The exploration of the bioactive potential of bovine pericardium membrane combined with 120 kDa hyaluronic acid is an innovation in the development of biomaterials for tissue regeneration. Collagen-based biomaterials, such as bovine pericardium membranes, have good mechanical properties and high biocompatibility, but still have limitations in supporting more effective cell adhesion and proliferation [23,24]. Therefore, this study explores the modification of bovine pericardium membranes by incorporating low-molecular-weight hyaluronic acid to alter surface characteristics, particularly hydrophilicity, which is relevant to tissue regeneration applications. The results of this study show that the combination of bovine pericardium membrane with hyaluronic acid molecular weight of 120 kDa alters the physicochemical and structural characteristics of the membrane, as evidenced by physicochemical characterization and SEM and FTIR analyses. SEM and FTIR analyses confirmed changes in surface morphology as well as chemical interactions indicating increased hydrophilicity and material stability after modification [25,26].

The increase in oxygen content observed with higher hyaluronic acid (HA) concentrations can be attributed to the intrinsic chemical structure of HA, which is rich in hydroxyl (–OH) and carboxyl (–COO^-^) functional groups. These oxygen-containing moieties contribute directly to the elevated oxygen signal detected by surface elemental analysis. In addition, the incorporation of HA enhances the hydrophilicity of the membrane surface, facilitating greater water adsorption and hydration layer formation. This hydrated interface is known to promote favorable cell–material interactions, including improved protein adsorption and cell attachment [27,28]. From a biological perspective, increased surface oxygen and hydrophilicity are associated with enhanced fibroblast adhesion and spreading, which are critical for early tissue integration. Regarding long-term biomaterial performance, the presence of oxygen-rich functional groups may support sustained biocompatibility by maintaining a moist microenvironment and reducing unfavorable protein denaturation, thereby potentially contributing to stable cell responses without inducing cytotoxic effects.

The MTT assay demonstrated that all hyaluronic acid–modified bovine pericardium membranes supported fibroblast viability and did not exhibit cytotoxic effects. These findings indicate that the modified membranes are biocompatible in vitro and capable of supporting cellular viability, which is an important prerequisite in the development of regenerative biomaterials [29,30]. In addition, XRD tests show that this combination retains an amorphous pattern that may contribute to material flexibility and compatibility with biological tissues. No statistically significant differences in fibroblast viability were observed among the tested hyaluronic acid concentrations.

The analysis of SPSS data showed that although slight variations in mean viability were observed among HA concentrations, statistical analysis confirmed no significant differences between groups. The results of the normality test with Kolmogorov-Smirnov and Shapiro-Wilk showed that the data had a normal distribution with a significance value above .05 [31,32]. The ANOVA test showed no significant difference between the treatment groups (p > 0.05) and the Tukey HSD test further confirmed that differences in viability among groups were not statistically significant. Therefore, variations in mean viability values should be interpreted descriptively and within the limitations of in vitro analysis [33,34].

Although the results of this study show potential in medical applications, there are still some challenges that need to be addressed. One of the main challenges is the long-term stability of materials and the potential for degradation in the biological environment [35]. In addition, further research is needed to evaluate the effectiveness of this combination in vivo models to ensure that these materials can actually be used safely and effectively in clinical therapies. Other factors such as the immune response and long-term biological interaction also need to be considered [36].

Overall, this research contributes to the development of biomaterials for tissue regeneration by providing in vitro evidence of the physicochemical characteristics and biocompatibility of bovine pericardium membranes modified with low-molecular-weight hyaluronic acid. However, further experimental and clinical investigations are required to determine the functional performance and clinical relevance of this material in tissue engineering applications [37,38].

## 5 Conclusion

This study demonstrated that bovine pericardium membranes modified with hyaluronic acid (120 kDa) exhibited altered physicochemical and structural characteristics, as evidenced by SEM and FTIR analyses. The characterization results indicated increased surface hydrophilicity following hyaluronic acid incorporation. Fibroblast viability assessment using the MTT assay showed that all hyaluronic acid–modified membranes supported cellular viability and did not exhibit cytotoxic effects.

Within the limitations of this study, no statistically significant differences in vitro biocompatibility were observed among the tested hyaluronic acid concentrations. However, further research, including in vivo trials and long-term evaluations, is still needed to ensure the effectiveness and safety of these biomaterials in clinical applications.

## Submission declaration

We declare that this work has not been previously published, except in the form of a preprint, abstract, published lecture, academic thesis, or registered report, and is not currently under review in any other journal. All authors have approved the submission and publication of this manuscript and have obtained approval from the relevant authorities at the institution where the research was conducted. We hope this manuscript will be considered for publication and are prepared to provide any necessary clarifications or revisions.

## Ethics approval and consent to participate

Ethical approval for this study was obtained from the Health Research Ethical Clearance Commission, Faculty of Dental Medicine, Universitas Airlangga (Approval Number: 0182/HRECC.FODM/II/2025). Ethical approval covered the use of biological materials and cell cultures in accordance with the study objectives and procedures. As this study was conducted entirely in vitro, informed consent from human participants was not applicable.

## Funding sources

The authors confirm that this research was conducted without any financial support from funding agencies, commercial organizations, or non-profit institutions. No external grants or sponsorships were received for the study.

## CRediT authorship contribution statement

**Dwi Wahyu Indrawati:** Conceptualization, Data curation, Formal analysis, Writing – original draft. **Ernie Maduratna Setiawatie:** Methodology, Supervision, Validation. **Retno Pudji Rahayu:** Investigation, Methodology, Supervision, Validation. **Rizky Briliant Syah Manurung:** Formal analysis, Writing – review & editing. **Mohammed Ahmed Aljunaid:** Resources, Software, Visualization.

## Declaration of competing interest

The authors declare that they have no known competing financial interests or personal relationships that could have appeared to influence the work reported in this paper.

## Data Availability

Data will be made available on request.
