# Supplementary material for: Exploring the bioactive potential of bovine pericardium membrane combined with hyaluronic Acid: characterization and cellular viability analyses
Source: Biochem Biophys Rep. 2026 Feb 6;45:102490. doi: 10.1016/j.bbrep.2026.102490 (PMC12905706; doi:10.1016/j.bbrep.2026.102490)
Supplement: Multimedia component 1 [file mmc1.pdf]

UNIVERSITAS AIRLANGGA FACULTY OF DENTAL MEDICINE
HEALTH RESEARCH ETHICAL CLEARANCE COMMISSION

ETHICAL CLEARANCE CERTIFICATE

Number : 0182/HRECC.FODM/II/2025

Universitas Airlangga Faculty Of Dental Medicine Health Research Ethical Clearance Commission has studied the proposed research design carefully, Declared to be ethically appropriate in accordance to 7 (seven) WHO 2011, and therefore, shall herewith certify that the research entitled :

"Exploring the Bioactive Potential of Bovine Pericardium Membrane Combined with 1.0% 1300 kDa Hyaluronic Acid: Characterization and Cellular Viability Analyses"

Principal Researcher : **DWI WAHYU INDRAWATI, drg., SH., M.Kes.,Sp.Perio**

Unit/Institution/Place of Research : - Research Center Faculty of Dental Medicine, Universitas Airlangga

CERTIFIED TO BE ETHICALLY CLEARED

Surabaya, February 10, 2025
Chairman,

PANITIA KELAIKAN ETIK FAKULTAS KEDOKTERAN GIGI UNIVERSITAS AIRLANGGA

Prof. Dr. TAMARA YUANITA, drg., MS., Sp.KG(K)
Official No. 196006251986012002
